# The feasibility of establishing parent support groups for children with congenital Zika syndrome and their families: a mixed-methods study

**DOI:** 10.12688/wellcomeopenres.16839.1

**Published:** 2021-06-17

**Authors:** Tracey Smythe, Veronika Reichenberger, Elisa María Pinzón, Isabel Cristina Hurtado, Luisa Rubiano, Hannah Kuper

**Affiliations:** 1 London School of Hygiene & Tropical Medicine, London, UK; 2 Secretaria deSalud de Valle del Cauca, Cali, Colombia; 3 Fundación Casa GAMI, Cali, Colombia

**Keywords:** Zika, disability, microcephaly, early intervention, congenital Zika syndrome, family, caregiver, Colombia

## Abstract

**Background:** The Zika epidemic highlighted gaps in health and social care services for parents of children with developmental disabilities. We aimed to evaluate the feasibility of a 10-week support group, ‘Juntos’, as a community-based intervention for parents of children with congenital Zika syndrome (CZS) in Colombia.

**Methods:** Two facilitators delivered ‘Juntos’ to four groups of 8-10 caregivers. One researcher observed each group. Data were collected from: observation notes from 40 sessions, focus group discussions held after each session, pre- post intervention questionnaires with 34 caregivers, and semi-structured interviews conducted with four facilitators, 12 caregivers and three stakeholders. We used the Bowen framework in data analysis.

**Results:** Acceptability and demand for the intervention were high. ‘Juntos’ was largely delivered with fidelity. Practicality was facilitated by providing transport costs and selecting convenient locations. Adaption requirements included additional organisational and social media support. Community health worker training may support integration and the established groups could facilitate programme expansion; however, participants perceived lack of prioritisation as a limitation. Participants’ knowledge and confidence to care for their child improved after programme enrolment.

**Conclusion:** Acceptability, demand and practicality of ‘Juntos’ is high. Yet implementation is challenged by existing health systems gaps in support of children with CZS.

## Abbreviations

CZS: Congenital Zika Syndrome, ZIKV: Zika Virus, CP: cerebral palsy

## What this study adds

- A parent group intervention is likely feasible to deliver in Colombia.
- It provides a unique approach to harness knowledge, experience and skills of families.
- Parent groups improve care provision for children with CZS.
- Parent groups build peer support for families of children with CZS.

## Introduction

Zika virus infection during pregnancy can cause microcephaly and other congenital anomalies ^1^. Common features characterising congenital Zika syndrome (CZS) include spasticity, seizures, eating difficulties, irritability, ocular anomalies, hearing loss, and abnormal neuroimaging ^2, 3^. As of January 2018, Colombia reported 248 CZS cases to the Pan American Health Organization (PAHO) ^4^. These cases represent the tip of the iceberg of CZS, and there are an estimated 276,100 children under five years with developmental disabilities more broadly in Colombia (2016 estimate) ^5^.

Developmental disabilities, which include CZS, are a heterogeneous group of conditions that can impact on the development of children's function (e.g. sensory, cognitive, physical) and contribute to child morbidity ^6^. However, the impact of developmental disabilities extends beyond the functioning of the child, and includes social, economic, mental health and emotional wellbeing consequences for the child and the family ^7, 8^. Therefore, the child and family have complex needs, including medical, educational, psychosocial and livelihood support. Services to meet these needs require a twin track approach to (1) encourage inclusion of children with disabilities in mainstream early child development, and (2) offer targeted approaches to address disability-specific issues ^6^.

Currently, health responses for CZS have mainly focussed on clinical needs. Our needs analysis in Brazil demonstrated a support programme for families of children with CZS was relevant and needed and a parent group intervention to provide psychosocial support and improve the skills of caregivers to optimally care for their child may fill an important gap in the Zika response ^9^. We used evidenced-based approaches to develop the ‘Juntos’ intervention to address this gap ^10– 13^. ‘Juntos’ (meaning ‘together’ in Portuguese and Spanish) consists of 10-modular weekly group sessions offered over a three-month period, in the community. The programme includes participatory learning about a range of topics ( Table 1). The curriculum emphasizes practice, problem-solving, and peer support through the group structure, and minimises didactic interactions between allied health professionals and parent participants. Each session includes icebreaker activities, practical sessions and group discussions, and a psychological support component, and lasts approximately 3–4 hours. Programme material is available from www.ubuntu-hub.org.

**Table 1.** Juntos intervention module topics.

Module number and title	Topics covered
1: Introduction	About the programme Information about Zika and CZS How to find information Personal stories
2: Our child	Introducing your close family and friends Development milestones for young children Determining your child’s progress Managing irritability and crying
3: Positioning and moving	How to position children who need assistance How to assist children to learn to move
4: Eating and drinking	Feeding challenges Practical skills to address challenges for your child
5: Communication	Importance of communication Practical advice to help your child communicate
6: Play and early stimulation	Importance of play for children to develop and learn Early stimulation Making simple toys Inclusion of play in the family and broader community
7. Everyday activities	How to use everyday activities to help your child develop Managing seizures
8. Uniting our voices	Understand the context of disability rights Education Communicating with your health team Advocating
9. Our community	Who is in your community? Common barriers to inclusion Addressing negative attitudes and exclusion Social Activity
10. Next steps	Summing up Planning next steps for yourself and the group

The participatory sessions with 8–10 caregivers are conducted by two trained facilitators: a parent of child with developmental disability (‘expert mother’) and a health professional (physiotherapist, occupational therapist, speech and language therapist or nurse). The health professional facilitates the technical aspects of skill acquisition and practice of techniques, such as feeding positions. The expert mother helps to foster a participatory and equal atmosphere, and encourages the sharing of learning between caregivers. The therapists and expert parents complete a joint standardised five-day facilitators training programme to prepare them for the delivery of the intervention, which includes practice of how to facilitate small group discussions, how to teach a practical skill, and takes a rights-based adult learning approach to content delivery. A structured manual supports their delivery of the sessions and that a consistent approach is taken.

A feasibility study was undertaken from August 2017 to June 2018 with six parent groups in two locations of Brazil – Rio de Janeiro and Greater Salvador, Bahia ^10^. Satisfaction and acceptability were high and demand was expressed by participants, facilitators, co-ordinators and stakeholders ^11, 12^. A question remains of the feasibility in other settings. Consequently, the aim of the current study is to evaluate the feasibility of implementation of ‘Juntos’ in the context of Colombia.

## Methods

Ethics approval for the study was granted from the London School of Hygiene & Tropical Medicine (LSHTM) (No 15986 /RR/11098) and Comité de etica e investigacion Asistencia cientifica de Alta complejidad (CEIACAC) Bogota (No CEI-022-19).

A mixed-methods study was undertaken in Cali May 2019–February 2020 to assess feasibility across four Juntos groups.

### Participants

Participants were caregivers of children with CZS (for this study defined as any child with impairments that can be directly attributed to Zika) identified through the database from the Public Health Surveillance System by a search filter for molecular diagnosis of ZIKV. Authors BMP and ICH had access to the database. The impairments in children ranged from mild cognitive, communication or functional skill delay to severe delay in all three developmental categories. Inclusion was restricted to children aged six months to five years, living within one hour of where ‘Juntos meetings’ were held, for whom informed written consent was obtained from the carer. Consent was obtained for participation in groups and publication of results. Children were ineligible if they had a condition requiring inpatient treatment at the time of intervention delivery (e.g. pneumonia) or were in institutional care.

Four Juntos groups were established, each including 8–10 caregivers. Study size was informed by the previous study in Brazil ^13^. Each group met weekly for approximately four hours for ten sessions. The groups were facilitated by one expert mother (of a child with CZS) paired with one health professional (physiotherapist or nurse). The study site co-ordinators identified and selected the facilitators. The health professionals and expert mothers completed a joint standardised four-day facilitators training programme to prepare them to deliver ‘Juntos’. The facilitators training programme was undertaken in Cali with an experienced trainer from the Brazil study and a trainer from the UK team (TS). The training included an introduction to the Juntos programme, exploring the empowerment journey for caregivers and understanding the fundamentals of a participatory approach, practising group activities from the Juntos manual and facilitating a small group, self-reflection and peer feedback, safeguarding and protection, and the role of monitoring and evaluation of groups. The expert mother was paid the same as the facilitator therapist (approximately USD 400 per month). Transport costs were reimbursed for all facilitators and all participants.

### Feasibility framework

We used the Bowen framework to assess feasibility in this study ^14^, and therefore focussed on the following eight components: acceptability, demand, implementation, practicality, adaption, integration, expansion, and limited efficacy ( Table 2).

**Table 2.** The eight areas proposed by Bowen *et al.* (2009).

Area of focus	The feasibility study asks…
**Acceptability**	*“To what extent is a new idea, program, process or measure judged as suitable, satisfying, or attractive to program* *deliverers? To program recipients?”*
**Demand**	*“To what extent is a new idea, program, process, or measure likely to be used (i.e., how much demand is likely to exist?)”*
**Implementation**	*“To what extent can a new idea, program, process, or measure be successfully delivered to intended participants in* *some defined, but not fully controlled, context?”*
**Practicality**	*“To what extent can an idea, program, process, or measure be carried out with intended participants using existing* * means, resources, and circumstances and without outside intervention*? *”*
**Adaptation**	*“To what extent does an existing idea, program, process, or measure perform when changes are made for a new format* * or with a different population?”*
**Integration**	*“To what extent can a new idea, program, process, or measure be integrated within an existing system?”*
**Expansion**	*To what extent can a previously tested program, process, approach, or system be expanded to provide a new program * *or service?*
**Limited efficacy**	**“** *Does the new idea, program, process, or measure show promise of being successful with the intended population, even* * in a highly controlled setting?”*

### Data collection and management

Data were collected from participants, facilitators, key stakeholders and through our cost tracking tools. All data collection tools were cognitively tested for understanding and administered in Spanish by the research assistants.

Data included:

1.    Participant data

Research assistants administered paper-based questionnaires the week prior to commencing the first session and within a week of completing the final group session. Questionnaires were administered at the site of the group sessions and included the following items (see Appendix 1, *Extended data* ^15^):

- Socio-demographic characteristics of the child and caregiver (baseline only)
- Understanding and knowledge about the child’s condition by the caregiver
- Perceived unmet needs and main goals for the intervention by the caregiver
- The PedsQL™ Family Impact Questionnaire Module (Colombian Spanish version) ^16^. This is a Likert scale questionnaire to assess health related quality of life and family functioning. It includes 36 items across the eight dimensions of physical functioning, emotional functioning, social functioning, cognitive functioning, communication, worry, daily activities, and family relationships.
- The Cantril scale ^17^ for assessing subjective quality of life of caregiver and of child, implemented as a 10 point ladder. Respondents are presented with image of a 10 rung ladder, asked to imagine the best possible life for them being a 10, and the worst possible life being a 0. They are then asked to rate their own current lives on that 0 to 10 scale
- Review of goals achieved (endline only)
- Satisfaction (scored out of five for satisfaction with the content, organisation and facilitators) and qualitative reflections on the intervention (endline only)

Semi-structured interviews were undertaken with 2–3 purposively sampled participants per group in order to reflect a broad range of perspectives (e.g. gender, age, high attenders, low attenders) (see Appendix 2, *Extended data* ^15^). Interviews were undertaken in Spanish by the research assistants after the final session of each group at the study site. They used a topic guide focussed on satisfaction with, and perceived impact of, the group intervention. The interviews took between 45 and 60 minutes, and were audio recorded, transcribed and translated to English.

2.    Facilitator data

Semi-structured interviews with each facilitator (total = 4) using a topic guide focussed on the reflections and lessons learned, perception of participants’ engagement and impact of ‘Juntos’ (see Appendix 3, *Extended data* ^15^). Interviews were undertaken in Spanish by the research assistants at the study site within a week after the 10th session of the final group. The interviews took between 45 and 60 minutes, and were audio recorded, transcribed and translated to English.

3.    Key stakeholders data

Semi-structured interviews with the study team leaders in Cali, two project coordinators and a senior medical provider, using a topic guide that focussed on practical components of implementing the sessions, reflections on lessons learned and potential future expansion (see Appendix 4, *Extended data* ^15^). Interviews were undertaken over video call in Spanish by VR, a female visual anthropologist. The interviews took between thirty and forty-five minutes, and were audio recorded, transcribed and translated to English.

4.    Cost data

Weekly cost data were collected by LR and included room rental, equipment, transport and refreshments for participants and facilitators. Budgets were analysed and summary statistics used to establish an overall cost for delivery of the intervention, and cost per participant. 

The research assistants also observed the sessions, made notes on logistics (e.g. timeliness, equipment required), the environment created by facilitators (e.g. room set up), the response of participants (e.g. contributions and participation) and response of children (e.g. irritability, engagement).

### Data analysis

Data analysis was guided by the Bowen feasibility framework. Qualitative data were analysed deductively with a framework analysis approach ^18^. A social scientist fluent in English and Spanish (VR) coded the interview responses. Analysis was evaluated by research team (TS), to ensure that interpretations were credible and valid. Frequent discussions with the research team took place throughout the data analysis phase. We undertook a narrative synthesis of the findings and reported the results according to the consolidated criteria for reporting qualitative research (COREQ) ^19^, which is a 32-item checklist.

Quantitative data was analysed using descriptive and inferential statistics within a statistical package (STATA.16). Socio-demographic characteristics of the caregiver and child were descriptively compared through frequencies and percentages. On the PedsQL Generic Core Scales, items were reversed scored and linearly transformed to a 0–100 scale as recommended ^20^. T-tests were used to compare the PedsQL scores for families, and Cantril scale for both caregiver and child, before and after the intervention. The satisfaction of content, organisation and facilitator quality were compared and analysed descriptively.

During data synthesis, the research team sought to use quantitative data to explain and illustrate qualitative findings, and look for congruence and incongruence between qualitative and quantitative findings.

## Results

A total of 34 families were recruited and characteristics of enrolled caregivers and children with CZS are shown in Table 3. The number of families enrolled in the four groups ranged from six to 10 families.

**Table 3.** Sociodemographic and health data of participants.

A. Enrolled primary caregiver, n= 34	
Characteristic	N (%) or mean ± SD
Mean age	
15 – 20 years old	3 (9%)
21 – 25 years old	11 (32%)
26 – 29 years old	6 (18%)
30 – 39 years old	11 (32%)
40 – 60 years old	2 (6%)
61 years and above	1 (3%)
Sex	
Female	33 (97%)
Male	1 (3%)
Highest education	
Never attended school	3 (9%)
Primary school	13 (38%)
Junior school	0 (0%)
Senior school	13 (38%)
Job training	5 (15%)
University	0 (0%)
Ability to work in the previous month	
No	22 (65%)
Yes	12 (35%)
Housing situation	
Home owner/family owned	10 (29%)
Rented	23 (68%)
Other	1 (3%)
Relationship status	
Married or civil partnership	25 (74%)
Divorced/separated	2 (6%)
Widow	0 (0%)
Single/never married	7 (20%)
B. Enrolled child, n= 34	
Sex	
Female	19 (56%)
Male	15 (44%)
Mean age in months	26 months ± 11 months
History of convulsions	16 (47%)
Diagnosis of epilepsy	14 (41%)
Up to date immunisations	31 (91%)
History of diarrhoea in last two weeks	5 (15%)
History of fever in last two weeks	8 (24%)
History of rapid or difficulty breathing in last two weeks	2 (6%)

### Acceptability

‘Juntos’ was considered to be acceptable and highly valued by the participants in three key ways: (1) it provided the opportunity to engage with others in similar situations; (2) it targeted areas that caregivers believed they needed help with; and (3) the use of expert mothers facilitated greater understanding of session content and encouraged provision of mutual support.

Participants reported finding a shared social identity and valued the opportunity to share personal experiences and problem solve together with others who understood their circumstances.

“ *I shared with other moms and learned about more children with the same condition as my daughter.*” Participant 03
“ *Before we started I was always exhausted. I felt tired, depressed and bored. But now, what I have done here has helped me overcome these feelings because I have met other mothers. I know their issues, and that has helped me a lot.*” Participant 07

Participants recognised that the intervention was targeting areas that they believed caregivers needed guidance and support with:

“ *We all do the activities and want to learn together, these positions, the way to feed our child, learning to ask for what we need, these are all things that we did not know before and they are important for all of us.”* Participant 2

The use of an expert mother was appreciated beyond a shared social identity. Participants viewed her role as practical as “ *she knew how to explain things to us in a way we could understand much easier*.” Participant 02

This satisfaction with the intervention is supported by the endline survey, with average scores (out of five), of 4.44 for content, 4.83 for organization and 4.87 for facilitator quality. The acceptability of the intervention was also articulated by the facilitator therapists and expert mothers. All four facilitators believed that ‘Juntos’ could be helpful to parents of children with disabilities as an adjunct to rehabilitation services.

Challenges to acceptability occurred with regard to the specific focus on children with CZS. Participants spoke about how being able to engage with other caregivers in similar situations was an attraction, and provided additional opportunities for mutual support:

“ *I think that every child, even those with minor disabilities, has the right to this program, because we all need help*.” Participant 04
“ *There are possibilities for children with Down syndrome to be included, so that they can learn like I did*” Participant 15

Children with cerebral palsy and toxoplasmosis were therefore included in the third and fourth group. There was no concern from caregivers of children with CZS to inclusion of children with other disabilities. Conversely, caregivers of children with other disabilities did not raise concern of being included with children with CZS. The differences between children was viewed as another opportunity to learn together and support each other.

### Demand

Demand for the group intervention was demonstrated by the generally high attendance and was expressed by the participants in interviews. Participants reported that they had no other opportunities to learn from each other, or problem solve together. This perceived unmet need was voiced as follows:

“ *I met other parents who have the same situation as myself…I have no one to share with, and I looked forward to the sessions because I took home something new*." Participant 02
“ *It has changed my social life. I love all of the moms, it has been great for me. I have new friends now…I think it is their acceptance. Feeling accepted because they are going through, or have gone through, the same things that I have and sometimes I can even help them back.*” Participant 03

Rehabilitation providers and stakeholders valued the group environment as it helped to promote family empowerment, which was recognised as absent in routine care.

“ *We need a programme that can provide families with hope, this is why I was convinced that ‘Juntos’ had to come to Cali.*’ Stakeholder 01
“ *Many families face stigma, and not all our children have access to appropriate therapies – this is the most important part of Juntos, to help families to cope and to face life with a different perspective*.” Stakeholder 03

In addition, participants viewed ‘Juntos’ as an opportunity to address the need to integrate other family members and siblings in the care of their child with CZS:

“ *Her sister loves this place, and my nephews love it too. When they saw children with the same condition as A’s for the first time, they were astonished*!” Participant 03

Juntos was viewed as addressing the unmet needs of participants through discussions and learning with others who understood their circumstances, and the experience was underpinned by the importance of building hope and empowering participants to better care for their child and understand their rights. No issues around demand for ‘Juntos’ were identified.

### Implementation

Four ‘Juntos’ group interventions (each of 10 sessions) were successfully completed by four facilitators. The expert mothers and therapist facilitators were observed to deliver the intervention content (e.g. 10 module topics) and processes (e.g. participatory learning, adult learning techniques) with fidelity. The expert mothers enacted their expected role within the group and were observed to foster a participatory and egalitarian atmosphere. The therapist facilitators supported the technical aspects of skill acquisition, such as feeding positions.

Facilitators needed to build their confidence to deliver sessions, and this took more time in sessions that were seen to require more specialist intervention, in particular the positioning and moving module.

“ *I had a little more difficulty with the positioning and movement session because I am not a physiotherapist…. but in facilitating the rest of the sessions I felt good.*” Therapist facilitator 01
“ *I have knowledge, but this* [facilitating technical skills in positioning and moving] *wasn’t my strength.*” Expert mother 01
“ *At the beginning, the facilitation process was not easy, sometimes I had the tendency to be more of a teacher and educator, not a facilitator*.” Therapist facilitator 02

Challenge to implementation may occur due to attendance. Participants reported being limited by other caring duties and by the illness of their child:

“ *We stayed home twice because my daughter was sick... I made an appointment for a home visit and I waited, but they did not arrive. I could have come that time, but I did not know they would not arrive*.”
Participant 02

Over the three-month period, the majority of caregivers missed at least one session due to illness of their child. Group attendance had an average of seven participants per session, ranging from four to 10 participants. There was no evidence of attendance going down over time.

### Practicality

The practicality of the intervention was facilitated by holding the group sessions at a suitable location in the community, which was a community primary school that was conveniently placed for participants. In addition, transport costs to participants were appreciated and facilitated attendance:

‘ *The transportation allowance you gave us has helped us a lot. Without it, several of us would not be able to come here. We have great expenses to deal with, and we can’t work. Mostly our partners provide for us and our children, if we happen to have a partner. This situation is exhausting*.’ Participant 03

Practicality was limited by the timing of sessions and observation notes suggest that at times the logistics could be challenging, with sessions often starting behind schedule and some not being able to finish all of the content. Time management improved in later groups as facilitators became more familiar with the content and set up requirements. The group sessions were designed to be held over two to three hours, and typically took approximately four hours.

The costs for running the intervention in Colombia amounted to USD 440 per participant for ten sessions. This included costs of the facilitator training, salaries of facilitators, and all the group activities (space rental, materials, refreshments and transportation). With higher group numbers and reducing the facilitator training costs as programmes are rolled out, running local programmes can be decreased further still.

### Adaptation

There was adaptation in the language and photograph content of the intervention from the first two groups to the last two groups as fast track learning and adjustments were made. These adaptions were few and primarily related to language (appropriate translation from Portuguese to Spanish) and context specific imagery. A ‘WhatsApp’ group was established as a communication tool between participants by the facilitators after the first week and supported the emotional wellbeing of participants.

' *I sent the photo to the* [WhatsApp] *group, and it was wonderful*'. Participant 03
‘ *When the programme ended we continued the WhatsApp group, and before COVID-19 we were able to meet once a month at a mother’s house.*’ Participant 08

More practical support was needed from the study co-ordinator than anticipated, including: providing support to facilitators in organisation of logistics (e.g. travel, food and intervention materials) and giving extra instruction on how to run practical sessions in order to maximise their impact.

### Integration

Program co-ordinators and a senior paediatric specialist considered the feasibility of integration of Juntos with respect to: (1) the availability of strategic and technical policies at national and district level; (2) the potential for greater collaboration between the general health service delivery sector and the rehabilitation sector to promote disability inclusive-health.

The availability of two national policies on rehabilitation and disability supports the feasibility of task-sharing in interventions to address child disability in the community ^21, 22^. However, there are gaps in the health system for children with disabilities, which may result in limited opportunity for integration. Participants experienced these practical barriers to access care regularly:

“ *We already have issues with receiving therapy because they* [the therapists] *haven’t been paid. We wrote to the Procurador, to the Health Minister, to the Health Superintendent and we hope… we will have a final answer and our children won’t be left without their therapies*.” Participant 06
“ *They denied me making specialist appointments, my son still needs to do some tests, and there are still rehabilitation therapies he hasn't accessed. I didn't get devices that could help C's positioning, that would improve his quality of life.*” Participant 15

Identifying and lobbying those with both interest and influence will be important to promote the integration of parent support groups such as these into national and district level policies that may in turn facilitate action:

‘ *Propose to the mayor so that we can have permission to formalise a parent group collaboration*’ Expert mother 02

### Expansion

Factors that support or hinder potential expansion include policy, leadership (e.g. key government stakeholders or non-governmental organisations [NGOs]), available funding and perceived need.

National guidelines on community-based rehabilitation (CBR) ^21^ provide a framework that include community health and disability training for community health workers at district level. Implementation of these guidelines may enable strategic service delivery, such as inclusive parent group interventions, through community health programmes. Additionally, training programmes in the national strategic health planning focus on early detection and referral of developmental disabilities. ‘Juntos’ could support other services provided for children with developmental disabilities.

Alongside appropriate supervision, financial compensation for training of new tasks and delivery of parent groups was emphasized as a necessary step for improving task-sharing in community-based health services. Funding is also required for longevity and sustainability of parent support programmes, and collaboration between NGOs and government is required to adopt a systematic approach to service delivery. In the Colombian government's model of care, partnership with NGOs is common, given the limited capacity of government entities to respond to all public health needs. For example, the Casa GAMI Foundation in Cali has worked with health secretaries, Ministry of Health and the United Nations Population Fund (UNFPA) to strengthen the response to the prevention of perinatal transmission of HIV. The NGO provides mentorship for women living with HIV and their families and collaborated on the implementation of this study. The work of Casa GAMI and similar family centred NGOs should be strengthened by working nationally with The Women's Secretariat and the Institute of Family Welfare.

Expansion is facilitated by the perceived need and strength of the established groups, who have plans to lobby and to educate, and integrate with, the community:

“ *I would love for the program to be extended, or at least, I would like that we, the older ones, would not be excluded, because we already have some wisdom about this, we know and we can guide others.*” Participant 03
“ *We have contacted a politician, who said he can help us, collaborate with us; but he is interested in a larger group because his father had a disability, so he already knows the process…*” Participant 02
“ *In my neighbourhood there is a public park that is full of people on Saturdays and Sundays. We could take a group of mothers with their children there and let them play at the park while we also explain Microcephaly to people, … but explain that they are children who can blend with others. I think that would make people more conscious about our children.*” Participant 03

Participants perceive expansion to be limited due to lack of prioritisation:

‘ *Our children require much more care and one of them* [health workers] *always forget about us*’ Expert mother 02
‘ *We are also human beings and we feel, we have needs, we lack your support and please do feel persuaded to support us and not to isolate us*.’ Participant 03

The perceived limited health leader role was believed to be a hindrance to expansion. Stakeholders suggested that virtual training and government ownership of the programme may expand the programme, and additional suggestions included:

“ *This programme does not replace therapy, but therapy is complicated and we need to share the impact of this programme with government bodies. We will need ongoing structured supervision of the facilitators*”
Stakeholder 02

Practical mechanisms for expansion include NGO or government leadership, a ‘training of trainers’ programme and mechanism for mentoring and supervision of the facilitators, and a structure to identify and enrol children on an ongoing basis.

### Limited efficacy

This study was not designed or powered to detect impact of the intervention, but rather to identify potential domains to assess efficacy.

In total, 34 participants completed pre-course questionnaires (response rate = 100%) and 23 completed post-intervention questionnaires (response rate = 67.6%). There was no evidence for a difference in either total scores or subscales between baseline and endline ( Table 4).

**Table 4.** The PedsQL™ Family Impact Questionnaire Module.

Dimensions of PedsQL	Baseline * (95%CI)	Endline ^&^ (95%CI)	p-value (t-test)
Physical functioning	53 (42 - 63)	57 (48 - 65)	0.73
Emotional functioning	57 (45 - 69)	64 (53 - 75)	0.35
Social functioning	57 (45 - 68)	58 (50 - 65)	0.83
Cognitive functioning	74 (64 - 84)	65 (55 - 75)	0.22
Communication	55 (42 - 70)	66 (55 - 76)	0.23
Worry	49 (36 - 62)	47 (38 - 57)	0.86
Daily activities	49 (41 - 61)	56 (44 - 62)	0.41
Family relationships	59 (43 - 74)	65 (51 - 79)	0.53
**Parent HRQL Summary**	60 (51 - 69)	61 (53 - 70)	0.85
**Functioning Summary**	55 (42 - 68)	61 (50 - 66)	0.46
**Total score**	58 (47 - 69)	61 (51 - 70)	0.51

*n=23. HRQL, health-related quality of life; CI, confidence interval.

In the Cantril Scale measurement (ladder of life), there was an increase in mean score from 5.8 to 6.9 (out of 10) for the self-reflection on happiness of the caregivers (n=22, p=0.24), although this was not a statistically significant change. There was no change in the perceived happiness of the child (5.7 to 5.5; n=22, p=0.53).

Qualitatively, there were a number of positive reflections which emerged from the interviews. They relate both to improvement in a child’s skill, and to the knowledge and confidence of the participant to care for her.

‘ *B improved a lot. She now crawls, she can hold herself still and she understands when you speak to her. If I ask B if she wants something, she will shake her head to indicate yes or no. She has improved a lot*.’ Participant 01
“ *Let's see, most important for me?....the knowledge around the care of B. You learn many things when you come here, things that you did not know. You learn and you let off steam, about how you feel, about what you learned...it has helped me a lot and I can now teach others*” Participant 01

Participants observed that as their knowledge and confidence increased, time required to complete caring duties decreased, and they were able to focus on livelihoods:

“ *My life has improved because I have had the opportunity to make my desserts and corn tortillas. My family then sells these things, so I now have an income to help my family..*." Participant 14

However, efficacy was perceived to be limited by participants due to the uncertainty of predicting the future needs of children with CZS, and unclear challenges in the growth and development of their children. Stakeholders reported that health professionals were unaware of the lifelong effects and prognosis of CZS, in addition to being ‘ *unprepared emotionally and academically*’ to provide care, both immediate and lifelong, for these children. In addition, stakeholders perceived lack of practical and medical guidelines, and implementation of these guidelines, as being a limitation to the follow through of community health programmes for children with CZS. They made the point that even though a guideline has been regulated in a policy document, it may take several years until the guideline results in direct action.

**Figure 1.**
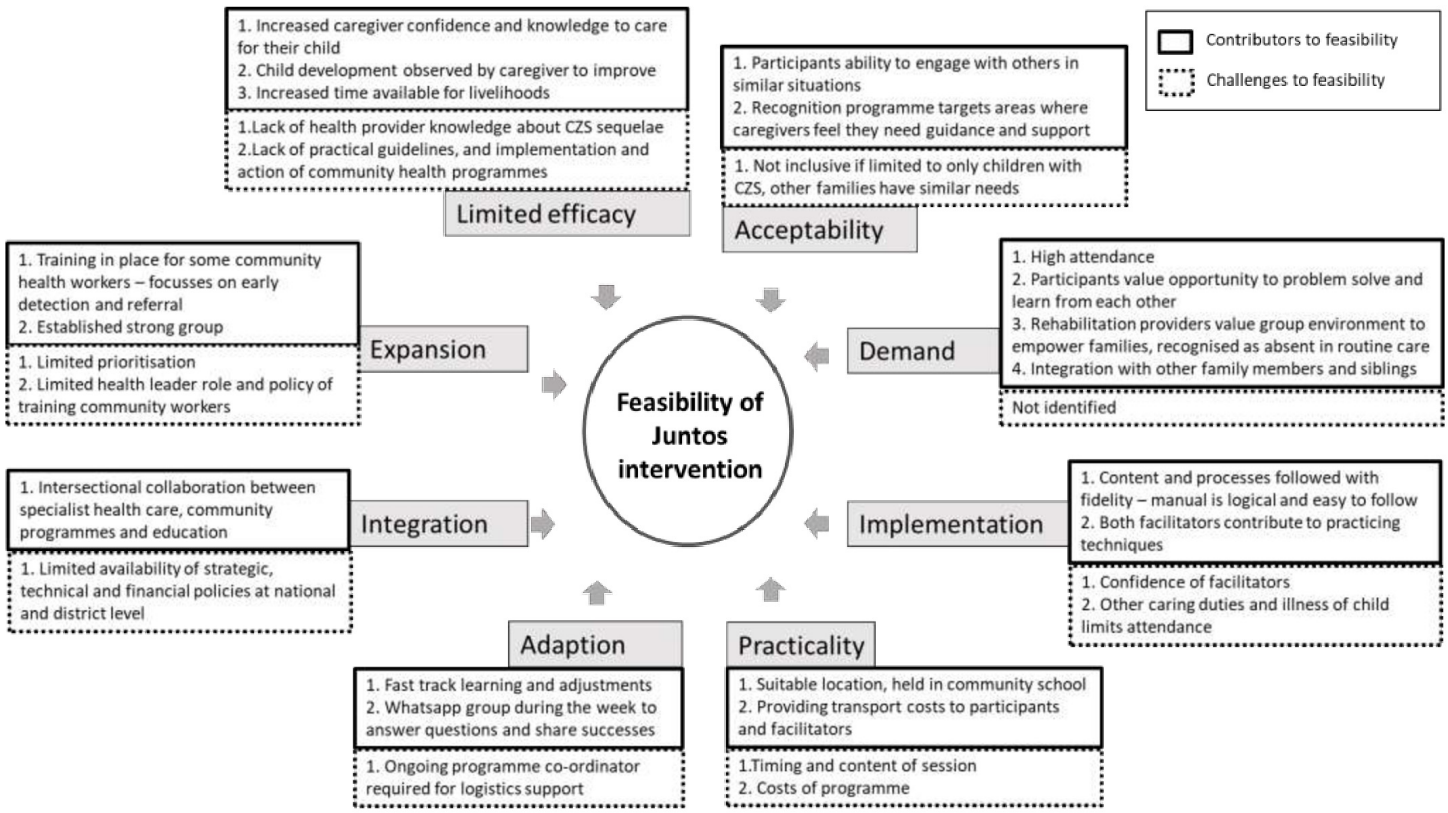
Contributing and limiting factors to feasibility of establishing parent groups for children with congenital zika syndrome (CZS) in Colombia.

The views among stakeholders and participants on factors that enable or hinder feasibility are shown in Figure 1.

## Discussion

This study showed that it was feasible to deliver the Juntos caregiver group intervention for families and children with CZS in Valle del Cauca, Colombia. There is demand for the intervention, the caregiver group structure and content are acceptable, the intervention was delivered with fidelity and was carried out at an existing community school location. Scale-up will require further supervision, policy and financial support. Efficacy of the intervention will need to be established through a larger-scale impact evaluation. However, the qualitative findings identified potential impacts, indicating that caregivers moved from being isolated, to finding strength and hope in shared experience and learning together. Participants also reported observing positive behavioural and developmental changes in their children. Parent-to-parent support was also reported to reduce levels of psychological distress and improve the ability to cope with being a parent of a child with a disability.

### Comparison to other studies

The Global Strategy for Women's, Children's and Adolescents' Health (2016-2030) recognises that people have a central role in improving their own health. Community participation is proposed to be central to achieving the global strategy’s objectives ^23^. Our findings contribute to the growing body of evidence of the importance of participatory peer learning. For example, a systematic review and meta-analysis of randomised controlled trials undertaken in Bangladesh, India, Malawi, and Nepal found that women’s groups working through participatory learning and action cycles were shown to be effective in improving maternal and newborn survival in rural settings in low-income countries ^24^, and ‘Juntos’ utilises similar participatory techniques. Findings regarding sustainability of women’s groups demonstrate that groups are more likely to be sustained if the group has local importance and members continue to acquire new knowledge ^25^. Similar to our findings, they also require leadership capacity, a unifying activity and a strong belief in the value of their meeting.

Focus on caregiver involvement and developing health workers’ skills may be critical to address the unknown long term outcomes of CZS, particularly where there are few health services. In addition, skills enhancement and training programs may provide an opportunity for quality assurance in service delivery. These findings are similar to those of a qualitative study in Ethiopia, India, Nepal, South Africa and Uganda that found task-sharing mental health services was perceived to be acceptable and feasible by stakeholders with ongoing structured supportive supervision at community and primary care-levels and provision of adequate training and compensation for health workers involved in task-sharing ^26^.

### Strengths and limitations

Study strengths include the use of a structured framework to assess the feasibility of the parent group intervention to address the needs of families of children with CZS in Colombia. The study included views of participants, facilitators and project co-ordinators who work within the health system of the Valle de Cauca. The study also has several limitations. First, the study may not be representative of all stakeholders within the health system and may not fully identify challenges in the areas of integration and expansion. However, the triangulation of findings between participants, expert mothers and facilitator therapists gives us confidence in our results although a full health system perspective is not included. Second, the study was conducted in one district; therefore, generalisation of these findings to other settings must be done with caution. Third, with a small sample size and the lack of comparison group it is not possible to draw conclusions with regards limited efficacy of the programme or the impact on families of children with different severities of functional impairment.

### Challenges and opportunities to scale

Participatory interventions have been challenged for being difficult to implement at scale. Our findings indicate three main challenges to future scale up. These are: (1) whilst community-based rehabilitation policies exist, they are not yet fully implemented; (2) an inadequate national support system for supervision and mentoring of health workers and expert mothers; and (3) limited central finance for implementation.

When considering future scale-up in Colombia, it is likely that adaptation of the facilitator training to include a greater number of expert mothers compared to the number of facilitator therapists may be required to account for contexts with fewer number of health workers. In addition, mothers may not have the capacity to travel as much or facilitate as many groups as the therapists. Scale up could be achieved through the availability of pairs of facilitators at community centres. Since task-sharing between health professionals and expert mothers has not yet started, the specific skills and competencies, as well as training, that fit the local health system and context need to be examined. Our findings show promise in developing and improving health professionals’ and expert mothers’ skills and competencies to task-share.

Several approaches may alleviate logistical and financial challenges, such as collaborating with community health programmes to improve logistical support for parent groups or implementation by NGOs. This study highlights the opportunity to integrate community participation in care for children with complex developmental disabilities and to work together to develop practical guidelines for implementing task-sharing.

## Conclusion

‘Juntos’ may be feasible under particular enabling conditions. Results highlight the imperative to plan and implement parent support programmes, inclusive of all children with developmental disabilities, as a component of a broader, integrated effort to strengthen the health system.

## Data availability

### Underlying data

Underlying data associated with this study will not be made freely available, as the small number of children with CZS makes data potentially identifying. Applications for access to the raw data for this study should be made via email to the corresponding author tracey.smythe@lshtm.ac.uk, outlining the purpose of the proposed analyses and the data requested. These applications will be reviewed by the LSHTM's data access committee, and if accepted, the requested data will be shared.

### Extended data

LSHTM Data Compass: Data collection tools for a study on establishing the feasibility of parent support groups for children with Congenital Zika Syndrome and their families in Colombia. https://doi.org/10.17037/DATA.00002245 ^15^.

This project contains the following extended data:

- 1-Pre-and-post_questionnaires_English.xlsx (Appendix 1: Participant pre and post questionnaire - English version)
- 1-Pre-and-post_questionnaires_Spanish.xlsx (Appendix 1: Participant pre and post questionnaire - Spanish version)
- 2-Participants_topic_guide_English.docx (Appendix 2: Topic guide focussed on satisfaction with, and perceived impact of, the group intervention - English language)
- 2a-Pre-intervention_topic_guide_Spanish.docx (Appendix 2a: Participant interview topic guide - Pre-intervention - Spanish language)
- 2b-Endline_topic_guide_Spanish.docx (Appendix 2b: Participant interview topic guide - Endline - Spanish language)
- 3-Facilitators_topic_guide_English.docx (Appendix 3: A topic guide focussed on the reflections and lessons learned, perception of participants’ engagement and impact of ‘Juntos’ - English language)
- 3-Facilitators_topic_guide_Spanish.docx (Appendix 3: A topic guide focussed on the reflections and lessons learned, perception of participants’ engagement and impact of ‘Juntos’ - Spanish language)
- 4-Key_stakeholders_topic_guide_English.docx (Appendix 4: A topic guide focussed on practical components of implementing the sessions, reflections on lessons learned and potential future expansion - English language)
- 4-Key_stakeholders_topic_guide_Spanish.docx (Appendix 4: A topic guide focussed on practical components of implementing the sessions, reflections on lessons learned and potential future expansion - Spanish language)

Data are available under the terms of the Creative Commons Attribution 3.0 International license (CC-BY 3.0).
