# Supplementary material for: The feasibility of establishing parent support groups for children with congenital Zika syndrome and their families: a mixed-methods study
Source: Wellcome Open Res. 2023 Jun 7;6:158. Originally published 2021 Jun 17. [Version 3] doi: 10.12688/wellcomeopenres.16839.3 (PMC10280024; doi:10.12688/wellcomeopenres.16839.3)
Supplement: Peer review history

# Peer review: Reviewer response for version 3

**DOI:** 10.21956/wellcomeopenres.21634.r59535

**Published:** 2023-06-12

**Authors:** Lidia B. Giúdici

**Affiliations:** 1 Academic, ALSEPNEO, CABA, Buenos Aires, Argentina; 2 Department of Health Promotion and Protection, Pedro de Elizalde Children′s Hospital, Buenos Aires, Argentina

The new version fulfills every requirements made previously. This kind of study should be disseminated through Latin America, and expanded to other conditions with special health care needs. Though every condition has its particular features, the need for help is similar in every caregiver.

Is the work clearly and accurately presented and does it cite the current literature?

Yes

If applicable, is the statistical analysis and its interpretation appropriate?

Partly

Are all the source data underlying the results available to ensure full reproducibility?

Partly

Is the study design appropriate and is the work technically sound?

Yes

Are the conclusions drawn adequately supported by the results?

Yes

Are sufficient details of methods and analysis provided to allow replication by others?

No

Reviewer Expertise:

Neurodevelopment; Growth; Follow-up of high risk newborns

I confirm that I have read this submission and believe that I have an appropriate level of expertise to confirm that it is of an acceptable scientific standard.

# Peer review: Reviewer response for version 1

**DOI:** 10.21956/wellcomeopenres.18574.r56611

**Published:** 2023-05-23

**Authors:** Hollie Hix-Small

**Affiliations:** 1 College of Education, Portland State University, Portland, Oregon, USA; 2 RISE Institute, Washington, District of Columbia, USA

The study examined the feasibility and effectiveness of a caregiver support group for children with congenital Zika syndrome in Columbia. The intervention consisted of sessions delivered over a period of 10 weeks by a trained parent and a professional.

 The sessions covered topics that are important to parents of children with disabilities. The structure of the intervention began with building a relationship between the facilitators and the caregivers, followed by enhancing caregiver knowledge about their child and their understanding child development. Subsequently, the sessions focused on topics that parents of children with disabilities typically prioritize, such as positioning, feeding, and play. The approach of embedding intervention opportunities into everyday, routine activities aligns with best practice in the field of Early Childhood Intervention.

 The authors of the study organized their findings using the eight categories outlined in Bowen's (2009) article. This approach to organizing the findings provides a useful framework for others who are examining facilitators and challenges in their own work. It allows for a systematic exploration of the factors that contribute to the success or difficulties encountered in implementing parent support groups for children with complex disabilities.

 The writing style is straightforward and clear, allowing readers to easily grasp the main points. However, there are areas that can be improved to enhance the clarity, replicability, and overall quality of the article.

 One aspect that requires clarification is the role of the research assistants. While the authors briefly mention their involvement, it would be helpful to have more detailed information regarding their specific responsibilities and contributions. In particular, further elaboration on how the research assistants were involved in the cognitive interviews would help readers better understand the research process and facilitate replication in future studies.

 Additionally, the article would benefit from additional information about the sessions. Although the module number and topics were listed, including additional information on the flow of the sessions and how the parent expert and professional worked together would provide readers with a clearer understanding of the intervention and its implementation. This would allow for better evaluation and potential replication.

 On page 6, the authors mention that children attended the sessions. However, up to that point, it was not explicitly clear that caregivers were allowed to bring their children to the sessions. Clarifying this aspect and stating explicitly that caregivers were encouraged to or could bring their children would enhance the article's clarity.

 Moreover, on page 7, a quote mentions that "Her sister loves this place," suggesting that siblings also attended the support group sessions. Additional information on the involvement of siblings, including their participation and roles within the sessions, would be valuable to readers seeking a comprehensive understanding of the study.

 Although the findings were not statistically significant, it is important to acknowledge that measuring the impact of parent support groups can be challenging. Perhaps it would be helpful for the authors to expand upon the limitations and potential reasons for the non-significant findings. This would provide readers with a better understanding of the complexities involved in assessing the effectiveness of such interventions.

 Regarding the writing style, minor edits are needed although they are not distracting to the reader (e.g., missing articles, tense, singular/plural, Oxford comma).

 While the data were not made freely available, the authors provide a suitable reason given the nature of the data and process for potentially obtaining a copy of the data.

 To summarize, this paper adds important knowledge, particularly for researchers and practitioners involved in designing feasibility intervention studies with similar populations. By addressing the mentioned areas for improvement, such as providing more information on the research assistants' roles, elaborating on the sessions, clarifying the inclusion of siblings, and addressing minor writing style issues, the article will provide an important contribution.

Is the work clearly and accurately presented and does it cite the current literature?

Yes

If applicable, is the statistical analysis and its interpretation appropriate?

Yes

Are all the source data underlying the results available to ensure full reproducibility?

Partly

Is the study design appropriate and is the work technically sound?

Yes

Are the conclusions drawn adequately supported by the results?

Yes

Are sufficient details of methods and analysis provided to allow replication by others?

Partly

Reviewer Expertise:

Early Childhood Intervention. Specifically, screening for developmental delays; professional development; integrated systems of care; infant and early childhood mental health; family-centered services; and self-determination.

I confirm that I have read this submission and believe that I have an appropriate level of expertise to confirm that it is of an acceptable scientific standard.

# Peer review: Reviewer response for version 1

**DOI:** 10.21956/wellcomeopenres.18574.r56181

**Published:** 2023-05-15

**Authors:** Lidia B. Giúdici

**Affiliations:** 1 Academic, ALSEPNEO, CABA, Buenos Aires, Argentina; 2 Department of Health Promotion and Protection, Pedro de Elizalde Children′s Hospital, Buenos Aires, Argentina

**General comment: **This is a very interesting study but the abstract is lacking some important information: 1) When did the Zika epidemic take place?; 2) When did they launch the intervention?; 3) Components of the intervention should be described; 4) Did the babies receive the disgnosis immediately after birth, before, or when? The population and the intervention should be better described in the abstract

**Abstract**

- *" **Methods:** Two facilitators delivered ‘Juntos’ to four groups of 8-10 caregivers. One researcher observed each group. Data were collected from: observation notes from 40 sessions, focus group discussions held after each session, pre- post intervention questionnaires with 34 caregivers, and semi-structured interviews conducted with four facilitators, 12 caregivers and three stakeholders. We used the Bowen framework in data analysis."*
  - **Comment** **: **Authors define “Juntos” as a “community-based intervention” for parents. The intervention should be explained in Methods Section
- **" *Results:*** *Acceptability and demand for the intervention were high. ‘Juntos’ was largely delivered with fidelity."*
  - **Comment** **: **We don´t know the interventions involved in “Juntos”. Again, we should have a brief description.
- **" *Conclusion:*** *Acceptability, demand and practicality of ‘Juntos’ is high. Yet implementation is challenged by existing health systems gaps in support of children with CZS*."
  - **Comment** **: **The abstract is not clear, as far as we don´t have a description of the intervention itself. I suggest a brief description of “Juntos”.
- ***"Keywords***
* Zika, disability, microcephaly, early intervention, congenital Zika syndrome, family, caregiver, Colombia"*
  - **Comments** **: **Microcephaly is not mentioned in the abstract. The time of the intervention is not described, so “early intervention” should be mentioned in the abstract.

**General comment**

 This study gives important information about the feasibility of an intervention-Juntos- delivered to parents of children with disabilities due to CZS. I consider of outmost importance the fact that parents were likely to receive in the group children with disabilities of diverse origins.

 This kind of intervention (for every child and families with special needs) have a high cost-effective impact and “goes” to the family and child.

**Introduction** **:**

 Clear and adequate

**Methods**

 There is a good description of the intervention, but as far as “early intervention” is a keyword for the authors, it is important to have a description of this as well.

**Results**

 Children´s included mean age was 26 months ±11 months old (then, the oldest were [taking into account 1SD] 37 months old; this is not “early intervention”. Please clarify or make clear how many children were included according to different age (months); e.g. less than 12 months; 12-24 months; 24-36 months, and older than 36 months. Further, did the authors find correlation between the child´s age and the effectiveness of the intervention?

Is the work clearly and accurately presented and does it cite the current literature?

Yes

If applicable, is the statistical analysis and its interpretation appropriate?

Partly

Are all the source data underlying the results available to ensure full reproducibility?

Partly

Is the study design appropriate and is the work technically sound?

Yes

Are the conclusions drawn adequately supported by the results?

Yes

Are sufficient details of methods and analysis provided to allow replication by others?

No

Reviewer Expertise:

Neonatology- Neurodevelopment

I confirm that I have read this submission and believe that I have an appropriate level of expertise to confirm that it is of an acceptable scientific standard, however I have significant reservations, as outlined above.
